# Supplementary material for: Epithelial GREMLIN1 disrupts intestinal epithelial-mesenchymal crosstalk to induce a wnt-dependent ectopic stem cell niche through stromal remodelling
Source: Nat Commun. 2025 Jun 4;16:5167. doi: 10.1038/s41467-025-60364-6 (PMC12137559; doi:10.1038/s41467-025-60364-6)
Supplement: Supplementary file 1 — Supplementary Information [file 41467_2025_60364_MOESM1_ESM.pdf]

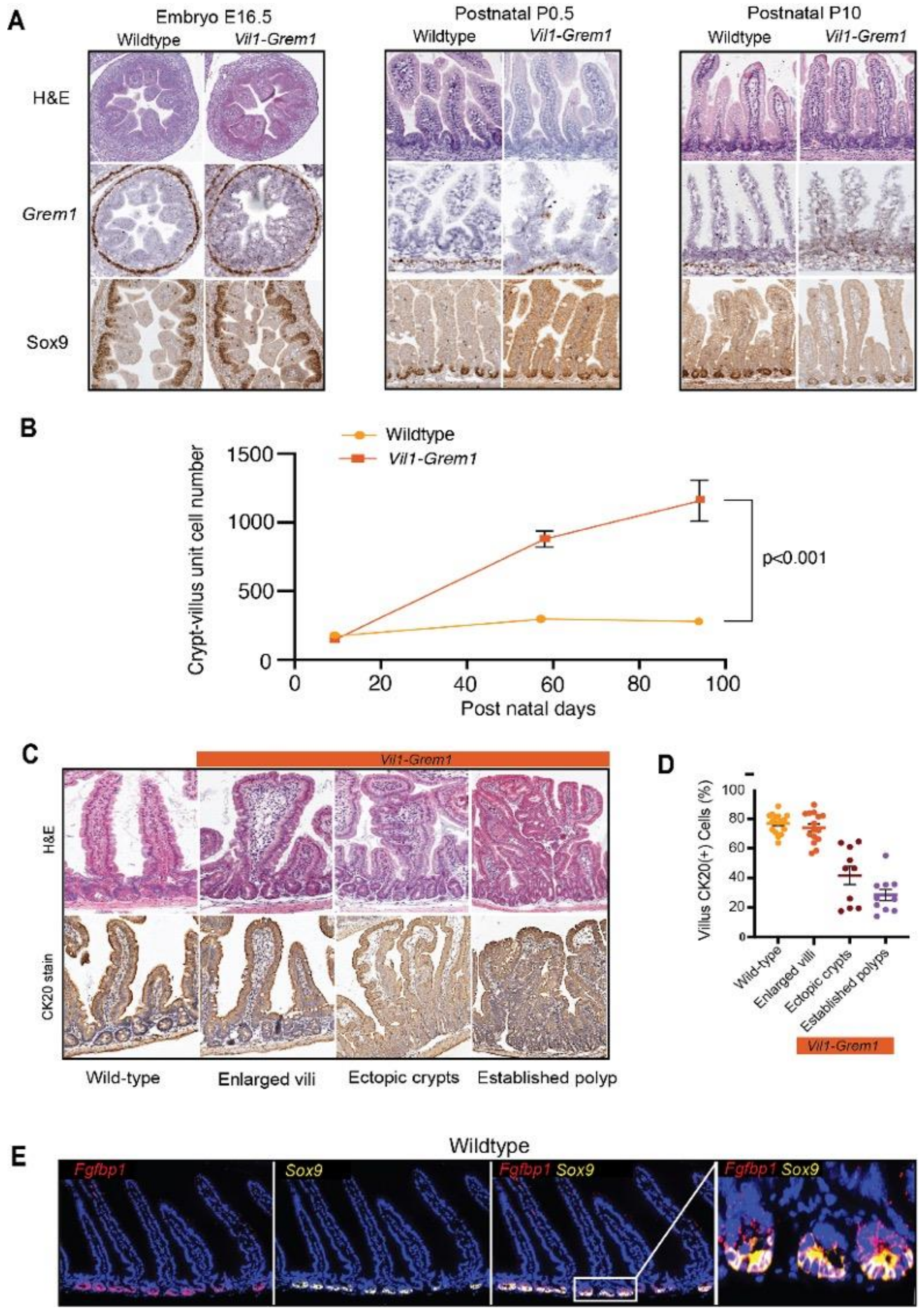


**Supplementary Figure 1. A.** Embryonic and postnatal expression of *Grem1* in wildtype and Vil-Grem1 animals. H&E, *Grem1* ISH and Sox9 IHC in varying embryonic and postnatal timepoints in wildtype and *Vil1-Grem1* animals (n ≥ 2). **B.** Crypt-villus unit cell number showing rapid expansion in *Vil1-Grem1* epithelial cell number from postnatal day 10 into adult mice (n=3 mice per group, ANOVA, p value<0.001). Data were ± s.e.m. **C.** CK20 staining of different morphological stages of lesion development in *Vil1-Grem1* mice shows reduced differentiation marker staining in ectopic crypts into established polyps (n=3 mice). **D.** Quantification of CK20 staining in different morphological stages of *Vil1-Grem1* mice polyp development (n=3 mice per group, ≥10 villi or polyps total, one way ANOVA with Dunnett post-hoc corrections, p values as stated). Data were ± s.e.m. **E**. Fluorescent co-ISH staining of *Sox9* and *Fgfbp1* in wildtype animals showing co-localisation in crypt base progenitor cell populations (n=5 mice). Scale bars 200µm. Source data are provided as a Source Data file.

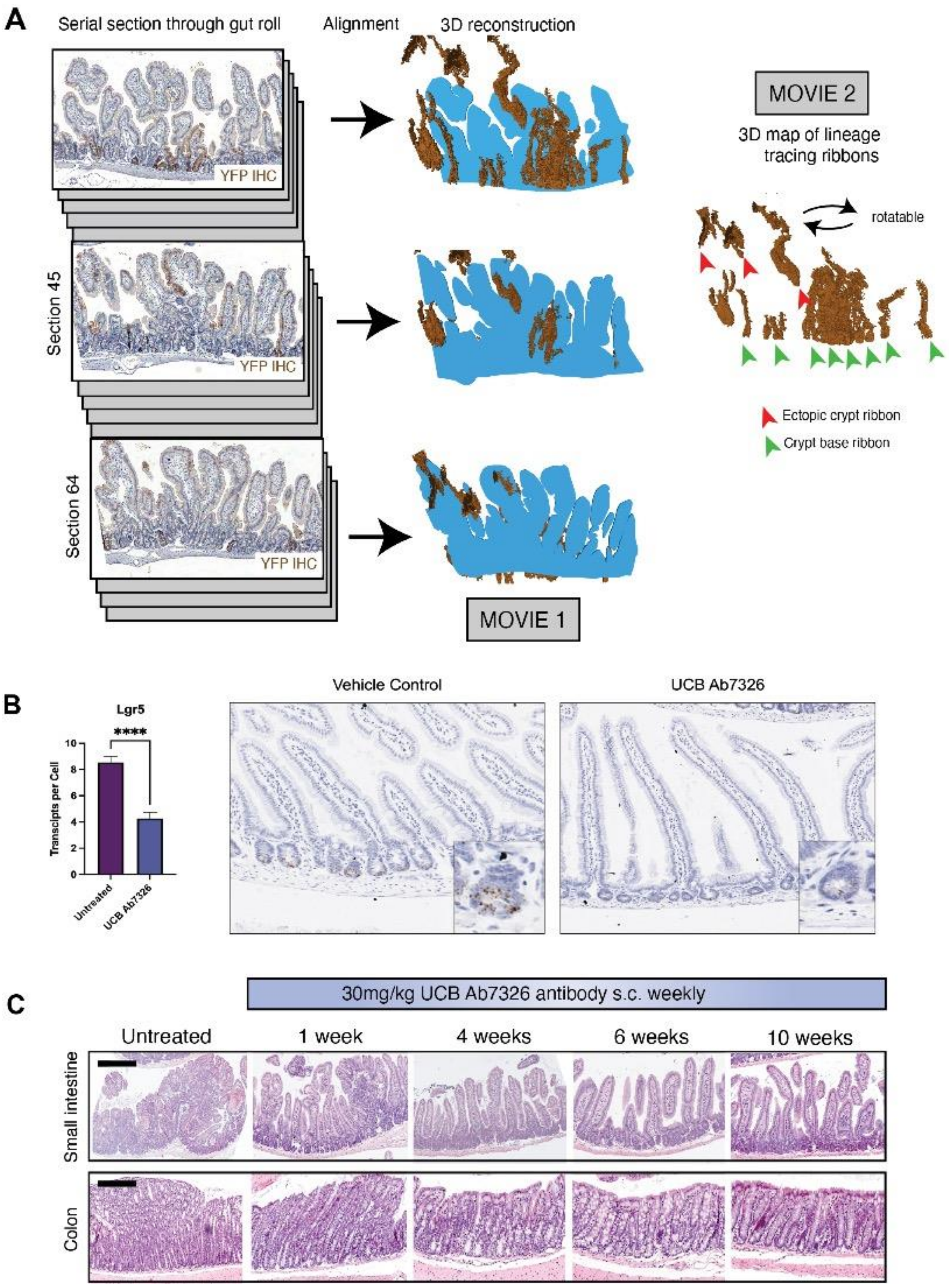


**Supplementary Figure 2. A.** 3D reconstruction to track lineage tracing ribbons through polyps. Careful serial sectioning through gut rolls with automated anti-YFP staining (brown) (n=1 whole mouse SI). Tissue alignment (HeteroGenius, Leeds, UK) and reconstruction of polyps allowed 3-dimensional tracking of lineage tracing ribbons (brown) to show that ectopic crypt ribbons (red arrowheads) were spatially distinct from those arising from the crypt base (black arrowheads). **B**. Reduction of expression of crypt basal stem cell markers in wildtype animals following UCB Ab7326 treatment. Representative *Lgr5* ISH images and quantification of *Lgr5* transcripts per cell (n=5 mice per group, two-tailed t test, p values****<0.001). Data were ±s.e.m. **C**. UCB Ab7326 treatment reverses *Vil1-Grem1* mouse polyposis phenotype**.** Representative H&E stains of colon and proximal small intestine of *Vil1-Grem1* animals following variable time treatment with UCB Ab7326 antibody (n=6 mice). Scale bars 200µm. Source data are provided as a Source Data file.

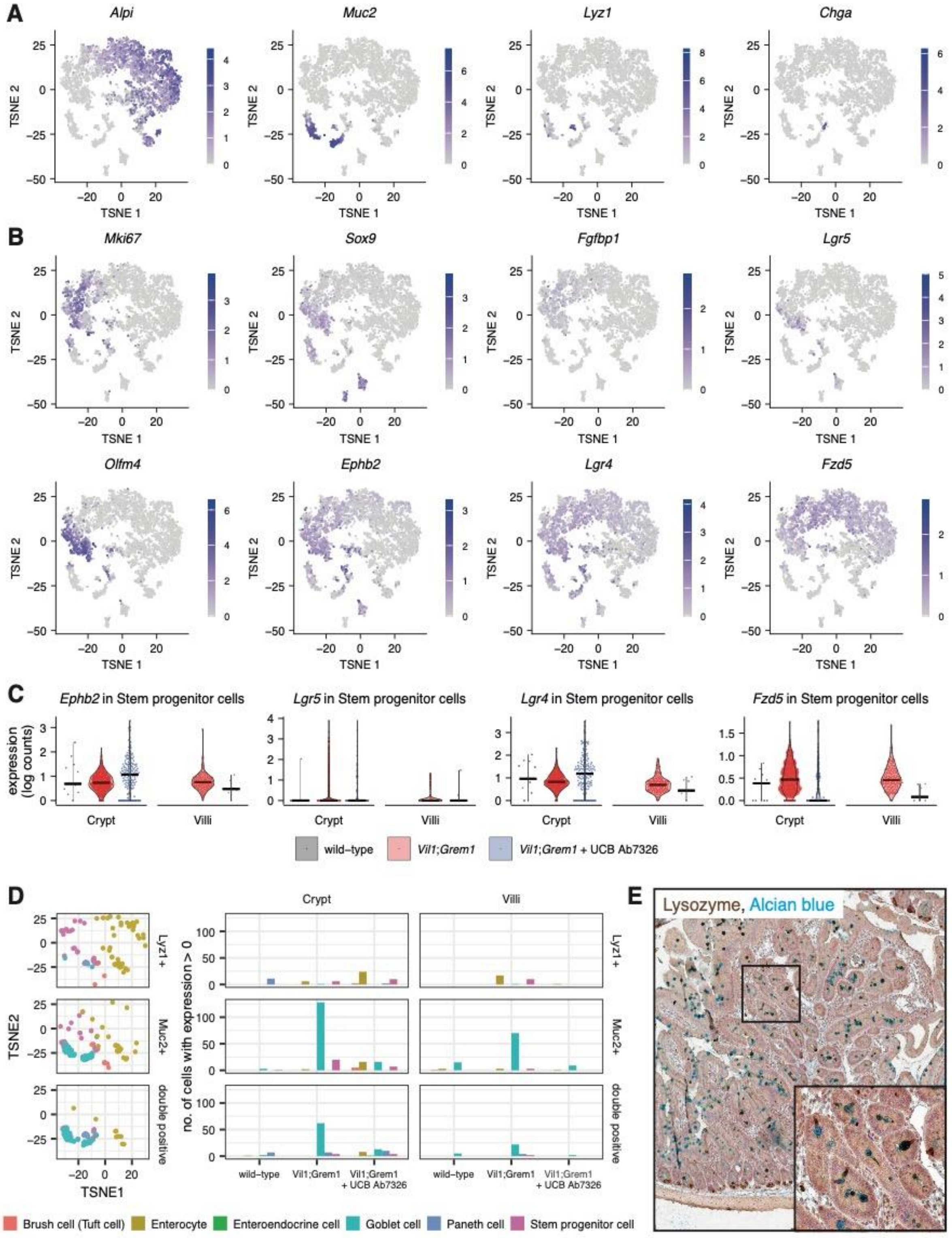


**Supplementary Figure 3. A.** T-SNE heatmaps of expression of representative differentiated intestinal epithelial cell markers *Alpi* (Enterocytes), *Muc2* (Goblet cells), *Lyz1* (Paneth cells) and *Chga* (Enteroendocrine cells), with expression shown as log(counts). **B.** T-SNE heatmaps of expression of intestinal stem cell markers, with expression shown as log(counts). **C.** Expression of intestinal stem cell markers in addition to those shown in Figure 3C in the stem progenitor cell population. **D.** t-SNE visualization of Lyz1+(Muc2-), Muc2+(Lyz1-), and Lyz1+Muc2+ double positive cells (assessed as cells with expression > 0), alongside quantification of these cells by cell type, mouse genotype/treatment, and tissue compartment. **E.** Representative image of Lyz1+Muc2+ double positive cells are illustrated in co-staining by Lyz1 IHC and Alcian blue of intestinal tissue from 100 day-old *Vil1*;*Grem1* mice (n=6).

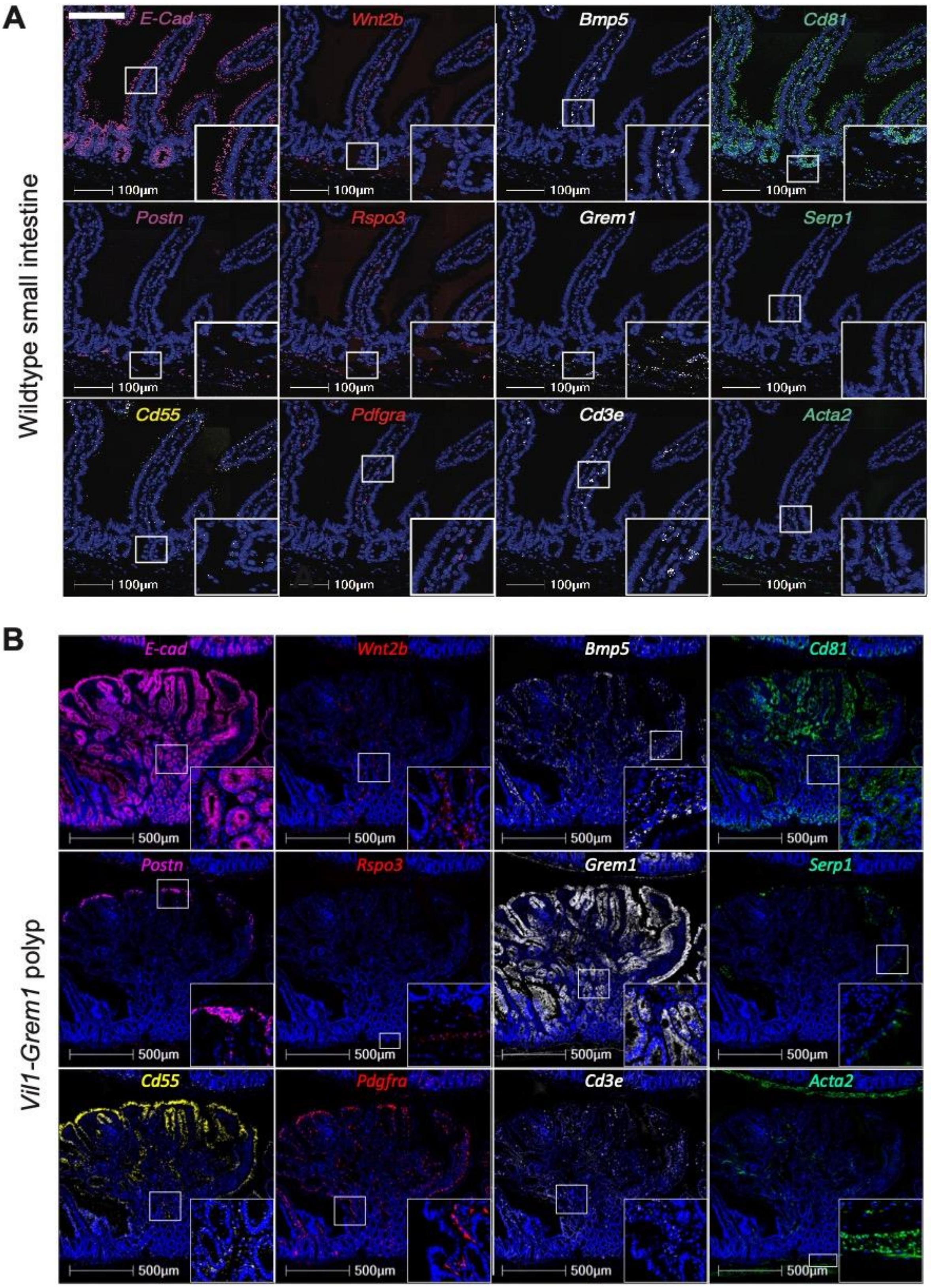


**Supplementary Figure 4. Multiplex ISH imaging of *Vil1-Grem1* mouse phenotype. A.** Intestinal fibroblast custom Hiplex panel - individual ISH stain of panel constituents in wild-type mice. **B**. Custom Hiplex staining of *Vil1-Grem1* polyps. Individual ISH probes as listed in colour legend (n=5 mice per geneotype).

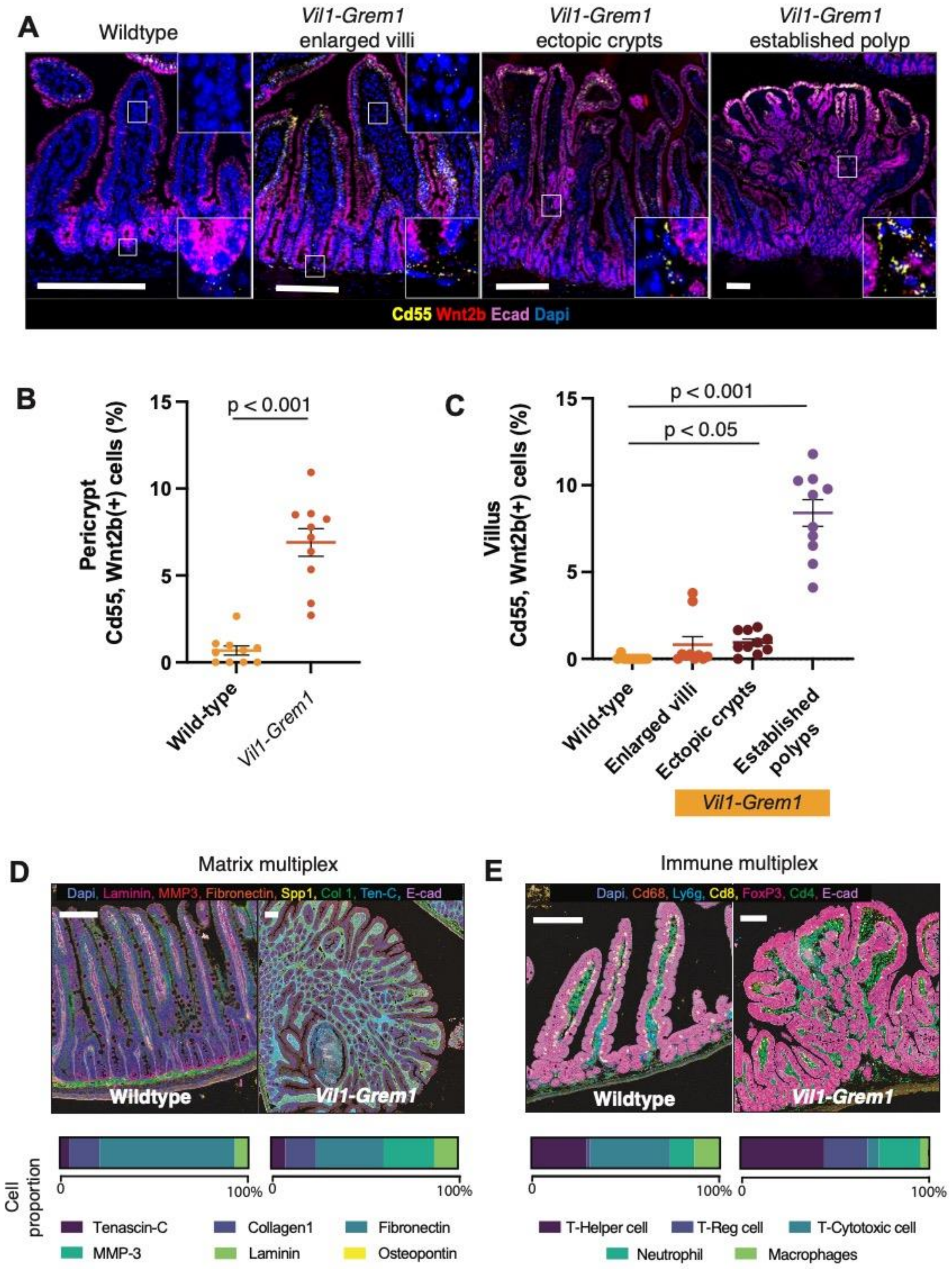


**Supplementary Figure 5. A.** Hiplex ISH staining of different morphological stages of lesion progression, showing expansion of the restricted pericrypt population of Cd55(+) Wnt2b(+) expressing fibroblasts up into the villi to surround ectopic crypts (n=5 mice). **B.** Quantification of Cd55(+) Wnt2b(+) fibroblasts in pericryptal regions of wild-type and *Vil1-Grem1* mice (n=5 mice per group, ≥10 crypts total, two-tailed t test, p values as stated) and **C.** in the villi of different morphological stage lesions in wild-type and *Vil1-Grem1* mice (n=5 mice per group, ≥10 villi or polyps total, one way ANOVA with Dunnett post-hoc corrections, p values as stated). Data were ± s.e.m. **D.** Representative images of multiplex IHC staining of matrix proteins in wildtype and *Vil1-Grem1* mice, with quantification of changes in protein proportion between genotypes (n=3) **E.** Representative images of multiplex IHC staining of immune cells in wildtype and *Vil1-Grem1* mice, with quantification of changes in cell proportion between genotypes (n=3). Scale bars 200µm. Source data are provided as a Source Data file.

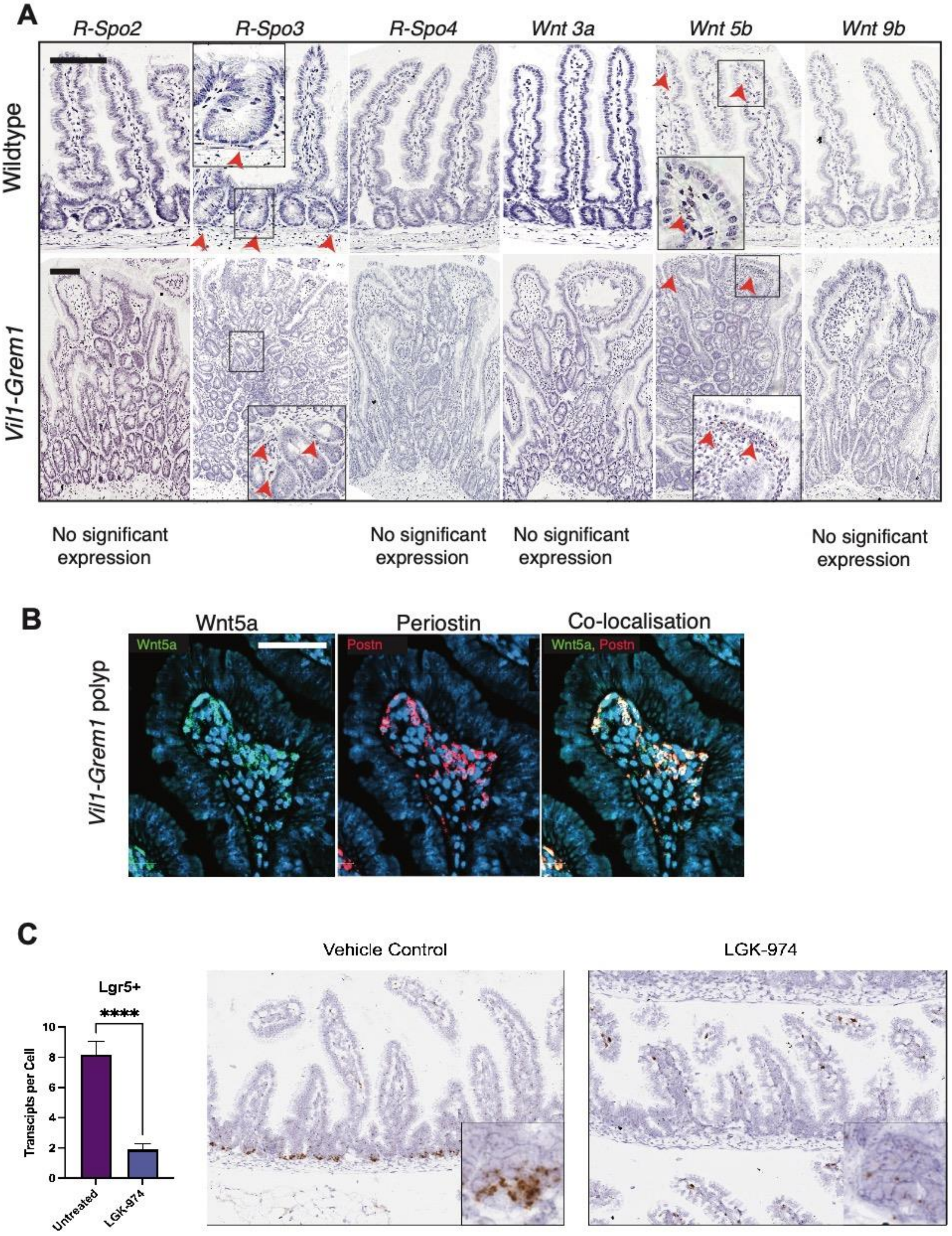


**Supplementary Figure 6. Additional wnt ligand staining and co-staining. A.** Comparison of staining of additional wnt ligands in wildtype and *Vil1-Grem1* mice (n ≥ 5 mice, scale bars 200µm.). **B.** Co-fluorescent ISH of Wnt5a and periostin at the periphery of *Vil1-Grem1* polyps (n=3 mice, scale bars 200µm). **C.** Reduction of expression of crypt basal stem cell markers in wildtype animals following LGK-974 treatment. Representative *Lgr5* ISH images and quantification of *Lgr5* transcripts per cell (n=5 mice per group, two-tailed t test, p values****<0.001). Source data are provided as a Source Data file.

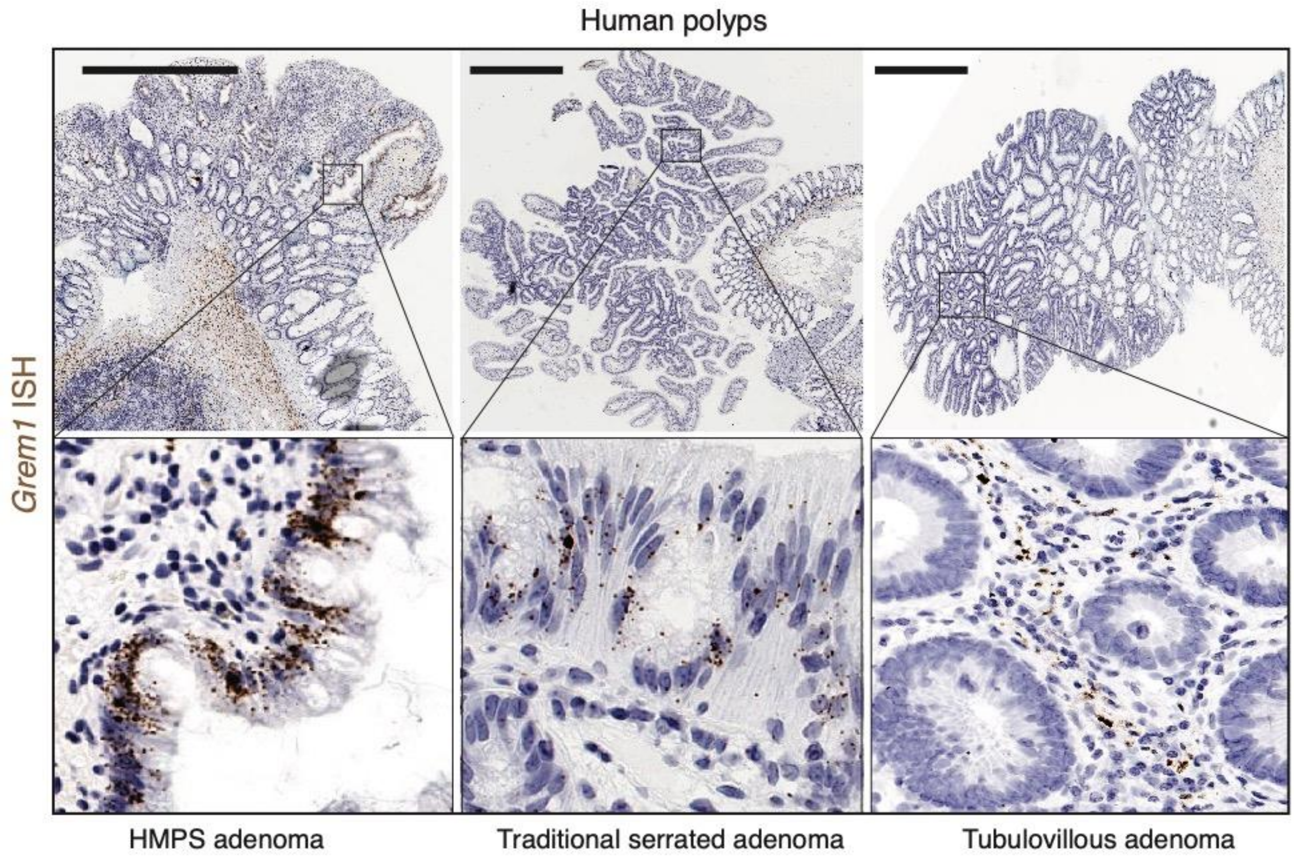


**Supplementary Figure 7. *GREM1* expression in HMPS and sporadic human polyps.** *GREM1* ISH (brown stain) in human polyps shows only stromal expression in sporadic tubulovillous adenomas (TVA) but marked epithelial expression in polyps from Hereditary Mixed Polyposis Syndrome (HMPS) and sporadic traditional serrated adenomas (TSA) (n ≥ 5 lesions per group). Scale bars 500µm.
