# Supplementary material for: Epithelial GREMLIN1 disrupts intestinal epithelial-mesenchymal crosstalk to induce a wnt-dependent ectopic stem cell niche through stromal remodelling
Source: Nat Commun. 2025 Jun 4;16:5167. doi: 10.1038/s41467-025-60364-6 (PMC12137559; doi:10.1038/s41467-025-60364-6)
Supplement: Supplementary file 2 — Description of Additional Supplementary Files [file 41467_2025_60364_MOESM2_ESM.pdf]

<u>Description of Additional Supplementary Files</u>

Supplementary Movie 1. Alignment and scrolling of serial sections through >120 day old *Sox9-CreER^{T2}; Rosa26^{YFP}; Vil1-Grem1* mouse small intestinal gut roll with anti YFP staining (brown) and automated false colorisation of tracing ribbons (green).

Supplementary Movie 2. Rotation of reconstructed polyp to show spatial segregation of crypt basal and ectopic crypt lineage tracing ribbons, to exclude interconnection of ribbons in three-dimensional space.
