# Supplementary material for: Epithelial GREMLIN1 disrupts intestinal epithelial-mesenchymal crosstalk to induce a wnt-dependent ectopic stem cell niche through stromal remodelling
Source: Nat Commun. 2025 Jun 4;16:5167. doi: 10.1038/s41467-025-60364-6 (PMC12137559; doi:10.1038/s41467-025-60364-6)
Supplement: Supplementary file 5 — Reporting Summary [file 41467_2025_60364_MOESM5_ESM.pdf]

nature portfolio

Corresponding author(s): Hayley Belnoue-Davis

Last updated by author(s): Apr 3, 2025

Reporting Summary

Nature Portfolio wishes to improve the reproducibility of the work that we publish. This form provides structure for consistency and transparency in reporting. For further information on Nature Portfolio policies, see our Editorial Policies and the Editorial Policy Checklist.

Statistics

For all statistical analyses, confirm that the following items are present in the figure legend, table legend, main text, or Methods section.

n/a	Confirmed	
☐	☒	The exact sample size (*n*) for each experimental group/condition, given as a discrete number and unit of measurement
☐	☒	A statement on whether measurements were taken from distinct samples or whether the same sample was measured repeatedly
☐	☒	The statistical test(s) used AND whether they are one- or two-sided<br>*Only common tests should be described solely by name; describe more complex techniques in the Methods section.*
☐	☒	A description of all covariates tested
☐	☒	A description of any assumptions or corrections, such as tests of normality and adjustment for multiple comparisons
☐	☒	A full description of the statistical parameters including central tendency (e.g. means) or other basic estimates (e.g. regression coefficient) AND variation (e.g. standard deviation) or associated estimates of uncertainty (e.g. confidence intervals)
☐	☒	For null hypothesis testing, the test statistic (e.g. *F*, *t*, *r*) with confidence intervals, effect sizes, degrees of freedom and *P* value noted<br>*Give P values as exact values whenever suitable.*
☒	☐	For Bayesian analysis, information on the choice of priors and Markov chain Monte Carlo settings
☒	☐	For hierarchical and complex designs, identification of the appropriate level for tests and full reporting of outcomes
☒	☐	Estimates of effect sizes (e.g. Cohen's *d*, Pearson's *r*), indicating how they were calculated

Our web collection on statistics for biologists contains articles on many of the points above.

Software and code

Policy information about availability of computer code

Data collection

No software was used for Data collection

Data analysis

For single-cell RNA sequencing: Cell demultiplexing and sequence alignment were performed with 10X Genomics Cell Ranger (v7.1.0) using the mm10-2020-A reference transcriptome. Counts due to ambient RNA molecules and random barcode swapping from the (raw) UMI-based scRNA-seq gene-by-cell count matrices were removed and empty droplets were filtered out using CellBender (v0.3.0). The processed single cell RNA-sequencing data were analysed in the R statistical environment using the scran (v1.32.0) and scater (v1.32.0) packages. Doublets were identified and excluded from the dataset using the scDblFinder (v1.18.0) package. Cells with mitochondrial gene expression percentages ≥ 15%, detected genes ≤ 200, and total UMI counts ≤ 1000 were excluded from the dataset. The data from each sample were normalized, merged, MNN-normalized, clustered using the Louvain method, and visualized by UMAP and T-SNE dimensionality reduction. Clusters of cells that showed distinct Epcam expression were selected from the dataset as epithelial cell clusters, and subjected to an initial semi-supervised cell type assignment against known mouse intestinal epithelial marker genes from the CellMarker 2.0 database using the algorithm in the scSorter (v0.0.2) package. Cell type annotations were further subjected to manual curation, validation, and confirmation using a range of mouse intestinal epithelial marker genes prior to further downstream data visualization and analysis.

For image analysis: IHC images were analyzed as follows: Positive cells were quantified using QuPath digital pathology software 42 (v0.2.3,), downloaded from https://QuPath.github.io/. Firstly, annotations of tissue areas were created for each sample with areas of folded tissue excluded to eliminate false positive signals. Cells were identified within QuPath using a custom algorithm established via stain separation using color reconstruction. Positive cell detection analysis was run to identify DAB positive cells and results reported as percentage of positive cells. Each annotation was manually verified for correct signal identification. For analysis of multiplex IHC, HiPlex ISH and dual ISH, HALO image analysis software (Indica Labs) was used to identify cell phenotypes, cell density analysis and mapping of cell phenotypes onto images. For 3D

reconstruction of lineage traced crypts in Vil1-Grem1-Sox9YFP polyps, images were aligned using HeteroGenius MIM with 3D Pathology AddOn (HeteroGenius, Leeds, UK).

For manuscripts utilizing custom algorithms or software that are central to the research but not yet described in published literature, software must be made available to editors and reviewers. We strongly encourage code deposition in a community repository (e.g. GitHub). See the Nature Portfolio guidelines for submitting code & software for further information.

Data

Policy information about availability of data

All manuscripts must include a data availability statement. This statement should provide the following information, where applicable:

- Accession codes, unique identifiers, or web links for publicly available datasets
- A description of any restrictions on data availability
- For clinical datasets or third party data, please ensure that the statement adheres to our policy

The single cell RNA-sequencing data has been made available in the BioStudies database (http://www.ebi.ac.uk/biostudies) under accession number E-MTAB-14360.

Research involving human participants, their data, or biological material

Policy information about studies with human participants or human data. See also policy information about sex, gender (identity/presentation), and sexual orientation and race, ethnicity and racism.

Reporting on sex and gender	n/a
Reporting on race, ethnicity, or other socially relevant groupings	n/a
Population characteristics	n/a
Recruitment	n/a
Ethics oversight	n/a

Note that full information on the approval of the study protocol must also be provided in the manuscript.

Field-specific reporting

Please select the one below that is the best fit for your research. If you are not sure, read the appropriate sections before making your selection.

☒ Life sciences ☐ Behavioural & social sciences ☐ Ecological, evolutionary & environmental sciences

For a reference copy of the document with all sections, see nature.com/documents/nr-reporting-summary-flat.pdf

Life sciences study design

All studies must disclose on these points even when the disclosure is negative.

Sample size	The sample size in this study was based on practical considerations and aligns with similar studies in the field. The majority of this work is based on tissue from mouse models. We used up to 13 mice per group, this ensured sufficient power to detect biologically meaningful effects while minimising animal use. Previous studies with similar designs have demonstrated robust findings with comparable sample sizes, which guided our choice here.
Data exclusions	No data was excluded from the analysis.
Replication	To ensure the reproducibility and robustness of our findings, all experiments were conducted with strict adherence to standardized protocols, including e.g., animal handling, dosing. We implemented consistent housing and handling conditions to minimize environmental variability among mouse groups. Additionally, we randomised mice to experimental groups to reduce selection bias. Where feasible, we included biological replicates by conducting experiments on mice from different litters to ensure that our findings were not limited to a single cohort. All data collection and analysis procedures were thoroughly documented to facilitate repeatability by other researchers.
Randomization	To ensure the robustness and reproducibility of our findings, randomisation was applied at multiple stages of the study. Mice were randomly assigned to experimental groups. This approach helped to minimize selection bias and ensured balanced representation across groups. During the experimental procedures, randomisation was also maintained for the order in which mice were handled and tested, reducing potential biases due to environmental or temporal factors.
Blinding	Blinding was not feasible in this study, as tissue sample preparation required group-specific handling. To minimize bias, we used strict, standardised protocols for all sample processing and data analysis. Additionally, data were analysed based on predefined criteria to ensure objectivity and consistency across groups.

Reporting for specific materials, systems and methods

We require information from authors about some types of materials, experimental systems and methods used in many studies. Here, indicate whether each material, system or method listed is relevant to your study. If you are not sure if a list item applies to your research, read the appropriate section before selecting a response.

Materials & experimental systems

n/a	Involved in the study
☐	☒ Antibodies
☒	☐ Eukaryotic cell lines
☒	☐ Palaeontology and archaeology
☐	☒ Animals and other organisms
☒	☐ Clinical data
☒	☐ Dual use research of concern
☒	☐ Plants

Methods

n/a	Involved in the study
☒	☐ ChIP-seq
☒	☐ Flow cytometry
☒	☐ MRI-based neuroimaging

Antibodies

Antibodies used	All antibodies used in this work are as follows: Anti-Ki-67 (D3B5) Rabbit mAb (Cell Signalling Technology, Cat#CS12202S); Anti-lysozyme Rabbit pAb (DAKO, Cat#EC3.2.1.17); Anti-mCherry (TdTomato) mouse mAb (Novus Bio, Cat#NBP1-96752); Anti-SOX9 Rabbit pAb (Sigma Aldrich, Cat#AB5535); Human/Mouse EphB2 Antibody (R&D, Cat#AF467; Phospho-SMAD1/5 (Ser463/465) Rabbit mAb (Cell Signalling Technology, Cat#41D10); Anti-β-Catenin Clone 14 (BD Biosciences, Cat#610154); HES1 (D6P2U) Rabbit mAb (Cell Signalling Technology, Cat#11988); Olfm4 (D6Y5A) XP® Rabbit mAb (Cell Signalling Technology, Cat#39141), anti-GFP/YFP Rabbit polyclonal Ab (ThermoFisher Scientific, Cat#A6455), anti-Cytokeratin 20 Rabbit polyclonal Ab (Abcam, ab118574).
Validation	All antibodies used in this study were validated through multiple approaches to confirm specificity and suitability. We first checked supplier-provided validation data and tested each antibody under the experimental conditions used in our study. We stained to verify target specificity and compared observed staining patterns to known expression profiles. These steps ensured the reliability and reproducibility of antibody-based detection in our experiments

Animals and other research organisms

Policy information about [studies involving animals](); [ARRIVE guidelines]() recommended for reporting animal research, and [Sex and Gender in Research]()

Laboratory animals	The mouse alleles used in this study in various combinations are as follows: Vil1-Grem1, Atoh1CreERT2, Villin-CreERT2 36, RosaCreERT2 37, Sox9CreERT2 38, Rosa26Grem1, Rosa26tdTom 39, Rosa26YFP 40, Lgr4fl/fl41. All on a C57BL/6 Background
Wild animals	We did not use wild animals
Reporting on sex	In this study, we did not report sex as a variable in our mouse experiments. The primary focus was on the effects of a specific treatments, where preliminary data indicated no significant sex differences in the outcomes assessed.
Field-collected samples	No field-collected samples
Ethics oversight	All procedures were carried out in accordance with Home Office UK regulations and the Animals (Scientific Procedures) Act 1986. All mice are housed in individually ventilated cages at the animal unit either at the Functional Genetics Facility (Wellcome Centre for Human Genetics, University of Oxford) or The Beatson Institute (Glasgow).

Note that full information on the approval of the study protocol must also be provided in the manuscript.

Plants

Seed stocks	n/a
Novel plant genotypes	n/a
Authentication	n/a
